# *mer*-[3-Phenyl-5-(2-pyridyl-κ*N*)-1,2,4-triazol-1-ido-κ*N* ^1^]bis­(2-quinolylphenyl-κ^2^ *C* ^1^,*N*)iridium(III) deuterochloro­form disolvate

**DOI:** 10.1107/S1600536810038596

**Published:** 2010-10-13

**Authors:** Peter G. Jones, Andreas Freund, Marc Debeaux, Wolfgang Kowalsky, Hans-Hermann Johannes

**Affiliations:** a Institut für Anorganische und Analytische Chemie, Technical University of Braunschweig, Postfach 3329, 38023 Braunschweig, Germany; b Labor für Elektrooptik am Institut für Hochfrequenztechnik, Technical University of Braunschweig, Postfach 3329, 38023 Braunschweig, Germany

## Abstract

In the title compound, [Ir(C_13_H_9_N_4_)(C_15_H_10_N)_2_]·2CDCl_3_, the coordination at iridium is octa­hedral, but with narrow ligand bite angles ranging from 74.85 (8) to 83.99 (8)°. The bond lengths at iridium show the expected *trans* influence, with Ir—N *trans* to C being appreciably longer than *trans* to N. The chelate rings are mutually perpendicular to a reasonable approximation [interplanar angles ranging from 77.79 (6) to 83.93 (7)°]. All ligands are approximately planar; the maximum inter­planar angles within ligands are *ca* 12°. One CDCl_3_ solvent molecule is severly disordered and was excluded from the refinement.

## Related literature

For the preparation of iridium complexes, see: Lamansky *et al.* (2001 ▶); Coppo *et al.* (2004 ▶). For the photoluminescent properties and color tuning of cyclo­metalated iridium complexes, see: Grushin *et al.* (2001 ▶); You & Park (2005 ▶); Stagni *et al.* (2008 ▶). For general background to organic light-emitting diodes (OLEDs), see: Hertel *et al.* (2005 ▶); Holder *et al.* (2005 ▶). For two recent related publications from our groups, see: Jones *et al.* (2010*a* ▶,*b* ▶).
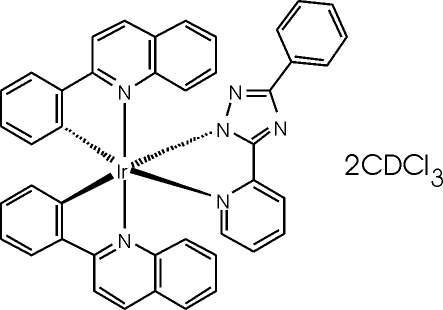


## Experimental

### 

#### Crystal data

[Ir(C_13_H_9_N_4_)(C_15_H_10_N)_2_]·2CDCl_3_
*M* *_r_* = 1062.67
Triclinic, 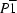

*a* = 9.1399 (3) Å
*b* = 12.4430 (5) Å
*c* = 17.6762 (6) Å
α = 81.493 (4)°
β = 81.509 (4)°
γ = 85.193 (4)°
*V* = 1962.41 (12) Å^3^
*Z* = 2
Mo *K*α radiation
μ = 3.86 mm^−1^
*T* = 100 K
0.25 × 0.20 × 0.05 mm

#### Data collection

Oxford Diffraction Xcalibur Eos diffractometer
Absorption correction: multi-scan (*CrysAlis PRO*; Oxford Diffraction, 2010 ▶) *T*_min_ = 0.739, *T*_max_ = 1.000
78670 measured reflections
9739 independent reflections
8125 reflections with *I* > 2σ(*I*)
*R*_int_ = 0.062

#### Refinement

*R*[*F* ^2^ > 2σ(*F* ^2^)] = 0.025
*wR*(*F* ^2^) = 0.048
*S* = 0.93
9739 reflections
487 parameters
134 restraints
H-atom parameters constrained
Δρ_max_ = 1.03 e Å^−3^
Δρ_min_ = −1.08 e Å^−3^

### 

Data collection: *CrysAlis PRO* (Oxford Diffraction, 2010 ▶); cell refinement: *CrysAlis PRO*; data reduction: *CrysAlis PRO*; program(s) used to solve structure: *SHELXS97* (Sheldrick, 2008 ▶); program(s) used to refine structure: *SHELXL97* (Sheldrick, 2008 ▶); molecular graphics: *XP* (Siemens, 1994 ▶); software used to prepare material for publication: *SHELXL97* and *PLATON* (Spek, 2009 ▶).

## Supplementary Material

Crystal structure: contains datablocks I, global. DOI: 10.1107/S1600536810038596/bt5363sup1.cif

Structure factors: contains datablocks I. DOI: 10.1107/S1600536810038596/bt5363Isup2.hkl

Additional supplementary materials:  crystallographic information; 3D view; checkCIF report

## Figures and Tables

**Fig. 1.**
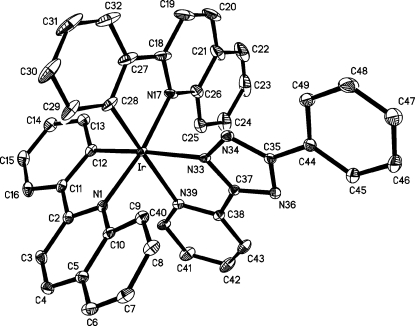
Structure of the title compound in the crystal. Ellipsoids represent 50% probability levels. Solvent molecules and hydrogen atoms are omitted for clarity.

## supplementary crystallographic information

### Comment

Electrophosphorescent materials based on iridium(III) have been one of the most important developments in the field of organic light-emitting diodes (OLEDs) because both singlet and triplet excitons can be harvested for light emission, giving OLEDs with theoretically 100% internal quantum efficiencies. Furthermore, iridium(III) complexes possess relatively short excited state lifetimes, high quantum efficiencies and remarkable colour tuning by modification of the ligand structures. The simple method of tuning the emission colour is to vary the combination of cyclometallating and ancillary ligands (*e.g.* acetylacetonate, picolinate or triazolate derivatives) coordinated to the iridium core. These heteroleptic complexes are particularly interesting as emitters for OLED applications. Quinoline-based iridium(III) complexes have proved to be especially efficient materials for red OLEDs. In this regard, we have synthesized and characterized the title compound, a new iridium(III) complex with 2-phenylquinoline as chromophoric ligands and 3-phenyl-5-(2-pyridyl)-1,2,4-triazole as ancillary ligand, and report here its crystal structure.

The structure of the title complex is shown in Fig. 1. It crystallizes with two molecules of deuterochloroform, one of which is severely disordered (see refinement details). The general features of the complex are similar to those of our other recent related structures (Jones *et al.*, 2010*a*,*b*). The coordination at iridium is octahedral, whereby the major deviations in angles arise from the restricted bite of the chelating ligands: N1—Ir—C12 79.82 (10), N17—Ir—C28 79.82 (12), N33—Ir—N39 74.85 (8)°. The bond lengths at iridium show the expected *trans* influence, with Ir—N33 and Ir—N39, 2.129 (2) and 2.196 (2) Å respectively, *trans* to C being appreciably longer than the mutually *trans* Ir—N1 2.084 (2) and Ir—N17 2.093 (2) Å. The interplanar angles between the chelate rings amount to 78.8 (1)° from the IrN_2_C_2_ ring to both IrNC_3_ rings, and 83.9 (1)° between the latter. Within the ligands, the interplanar angles between phenyl and quinoline are 11.8 (1) and 12.3 (1)°, whereas in the triazole ligand the pyridyl and phenyl rings subtend angles of 1.8 (1) and 11.0 (1)° respectively to the triazole ring.

### Experimental

A mixture of bis(2-phenylquinoline)-iridium(III)-µ-chloro bridged dimer complex (230 mg, 180 µmol), 3-phenyl-5-(2-pyridyl)-1,2,4-triazole (100 mg, 450 µmol) and potassium *tert*-butoxide (50 mg, 450 µmol) in dry dichloromethane (10 ml) and dry ethanol (3 ml) was stirred overnight at room temperature under nitrogen atmosphere. The solvent was removed under reduced pressure and the residue was purified *via* flash chromatography on silica gel (eluent: dichloromethane/acetone = 20:1, *R*_f_ = 0.31) to yield a red solid (115 mg, 39%). m.p. 326 °C.

^1^H NMR (CDCl_3_, 600 MHz): δ 8.13 (d, *J* = 9.0 Hz, 1H), 8.04–8.00 (m, 3H), 7.93–7.86 (m, 6H), 7.77 (d, *J* = 7.7 Hz, 1H), 7.52–7.47 (m, 3H), 7.29 (d, *J* = 8.9 Hz, 1H), 7.23 (dd, *J* = 7.6, 7.6 Hz, 2H), 7.18–7.14 (m, 2H), 7.11 (ddd, *J* = 7.9, 6.9, 1.0 Hz, 1H), 7.07–7.03 (m, 2H), 7.01 (dd, *J* = 7.3, 7.3 Hz, 1H), 6.95 (ddd, *J* = 7.2, 5.7, 1.3 Hz, 1H), 6.78–6.74 (m, 3H), 6.67 (dd, *J* = 7.0, 7.0 Hz, 1H), 6.46 (d, *J* = 7.5 Hz, 1H) p.p.m..

^13^C NMR (CDCl_3_, 150 MHz): δ 170.95, 170.01, 164.55, 162.54, 156.82, 151.88, 149.90, 148.24, 147.54, 146.98, 146.73, 146.61, 138.78, 138.31, 137.61, 136.01, 134.22, 133.17, 131.33, 130.06, 129.57, 129.47, 128.48, 128.14, 127.87, 127.49, 127.46, 127.26, 127.14, 126.53, 126.21, 126.05, 125.98, 125.54, 125.48, 123.23, 121.76, 121.65, 120.71, 116.99, 116.54 p.p.m..

EI—MS: m/*z* (%) = 822 (28) [*M*]^+.^, 601 (46) [M–C13H9N4]^+.^, 470 (4), 205 (100).

IR: = 3045 (w), 1604 (*s*), 1579 (*m*), 1544 (*m*), 1513 (*m*), 1460 (*m*), 1447 (*m*), 1421 (*m*), 1335 (*m*), 1288 (*m*), 1275 (*m*), 1242 (w), 1146 (*m*), 1070 (w), 1026 (*m*), 828 (*m*), 789 (*m*), 760 (*versus*), 724 (*s*), 695 (*s*), 640 (w), 569 (w), 539 (w) cm^-1^.

UV/Vis (CH_2_Cl_2_): λ (ε) = 446 (br. 4500), 337 (23600), 269 (58500), 227 (40300) nm.

Single crystals were obtained by evaporation from CDCl_3_ in an NMR tube.

### Refinement

Hydrogen atoms were included at calculated positions using a riding model with aromatic C—H 0.95, *sp*^3^-C—H 1.00 Å. The *U*(H) values were fixed at 1.2 × *U*_eq_(C) of the parent C atom. Anisotropic displacement parameters of the N and C atoms were restrained to have approximately equal components along mutual bonds (command DELU).

One deuterochloroform molecule is well ordered. However, a region of significant residual electron density could not be successfully interpreted in terms of the only possible solvent (CDCl_3_). The program SQUEEZE (as implemented in the *PLATON* system; Spek, 2009) was therefore used to remove mathematically the effects of this solvent. Values for the formula mass *etc*. are based on an assumed solvent content per asymmetric unit of one ordered and one squeezed CDCl_3_.

There are several peaks of 0.7–1.1 e Å^-3^ either *ca* 1 Å from the Ir atom, which may reasonably be attributed to residual absorption errors, or in the solvent region, corresponding to slight extra disorder or irregular displacement features.

### Crystal data

**Table:**

[Ir(C_13_H_9_N_4_)(C_15_H_10_N)_2_]·2CDCl_3_	*Z* = 2
*M**_r_* = 1062.67	*F*(000) = 1044
Triclinic, *P*1	*D*_x_ = 1.798 Mg m^−^^3^
*a* = 9.1399 (3) Å	Mo *K*α radiation, λ = 0.71073 Å
*b* = 12.4430 (5) Å	Cell parameters from 26610 reflections
*c* = 17.6762 (6) Å	θ = 2.2–30.8°
α = 81.493 (4)°	µ = 3.86 mm^−^^1^
β = 81.509 (4)°	*T* = 100 K
γ = 85.193 (4)°	Tablet, orange
*V* = 1962.41 (12) Å^3^	0.25 × 0.20 × 0.05 mm

### Data collection

**Table:**

Oxford Diffraction Xcalibur Eos diffractometer	9739 independent reflections
Radiation source: Enhance (Mo) X-ray Source	8125 reflections with *I* > 2σ(*I*)
graphite	*R*_int_ = 0.062
Detector resolution: 16.1419 pixels mm^-1^	θ_max_ = 28.3°, θ_min_ = 2.2°
ω–scan	*h* = −12→12
Absorption correction: multi-scan (*CrysAlis PRO*; Oxford Diffraction, 2010)	*k* = −16→16
*T*_min_ = 0.739, *T*_max_ = 1.000	*l* = −23→23
78670 measured reflections	

### Refinement

**Table:**

Refinement on *F*^2^	Primary atom site location: structure-invariant direct methods
Least-squares matrix: full	Secondary atom site location: difference Fourier map
*R*[*F*^2^ > 2σ(*F*^2^)] = 0.025	Hydrogen site location: inferred from neighbouring sites
*wR*(*F*^2^) = 0.048	H-atom parameters constrained
*S* = 0.93	*w* = 1/[σ^2^(*F*_o_^2^) + (0.019*P*)^2^] where *P* = (*F*_o_^2^ + 2*F*_c_^2^)/3
9739 reflections	(Δ/σ)_max_ = 0.003
487 parameters	Δρ_max_ = 1.03 e Å^−^^3^
134 restraints	Δρ_min_ = −1.07 e Å^−^^3^

### Special details

**Table:**

Geometry. All e.s.d.'s (except the e.s.d. in the dihedral angle between two l.s. planes) are estimated using the full covariance matrix. The cell e.s.d.'s are taken into account individually in the estimation of e.s.d.'s in distances, angles and torsion angles; correlations between e.s.d.'s in cell parameters are only used when they are defined by crystal symmetry. An approximate (isotropic) treatment of cell e.s.d.'s is used for estimating e.s.d.'s involving l.s. planes.Least-squares planes (*x*,*y*,*z* in crystal coordinates) and deviations from them (* indicates atom used to define plane)8.0107 (0.0039) *x* - 5.0559 (0.0100) *y* + 1.0694 (0.0169) *z* = 0.3793 (0.0119)* 0.0612 (0.0010) Ir * -0.0747 (0.0014) N1 * 0.0495 (0.0016) C2 * 0.0270 (0.0017) C11 * -0.0630 (0.0014) C12Rms deviation of fitted atoms = 0.05743.5166 (0.0086) *x* + 11.8000 (0.0045) *y* + 4.6463 (0.0182) *z* = 11.4217 (0.0048)Angle to previous plane (with approximate e.s.d.) = 83.93 (0.07)* -0.0639 (0.0011) Ir * 0.0668 (0.0016) C28 * -0.0280 (0.0019) C27 * -0.0526 (0.0019) C18 * 0.0777 (0.0015) N17Rms deviation of fitted atoms = 0.06022.1808 (0.0086) *x* + 0.5392 (0.0092) *y* + 17.4903 (0.0021) *z* = 5.6802 (0.0088)Angle to previous plane (with approximate e.s.d.) = 78.84 (0.08)* -0.0035 (0.0009) Ir * 0.0040 (0.0013) N33 * -0.0019 (0.0016) C37 * -0.0031 (0.0016) C38 * 0.0045 (0.0014) N39Rms deviation of fitted atoms = 0.00358.0107 (0.0039) *x* - 5.0559 (0.0100) *y* + 1.0694 (0.0169) *z* = 0.3793 (0.0119)Angle to previous plane (with approximate e.s.d.) = 78.79 (0.08)* 0.0612 (0.0010) Ir * -0.0747 (0.0014) N1 * 0.0495 (0.0016) C2 * 0.0270 (0.0017) C11 * -0.0630 (0.0014) C12Rms deviation of fitted atoms = 0.05748.1366 (0.0044) *x* - 4.6169 (0.0120) *y* + 2.7467 (0.0185) *z* = 1.1577 (0.0155)Angle to previous plane (with approximate e.s.d.) = 5.58 (0.11)* -0.0062 (0.0018) C11 * 0.0042 (0.0018) C12 * 0.0004 (0.0019) C13 * -0.0031 (0.0020) C14 * 0.0011 (0.0020) C15 * 0.0036 (0.0019) C16Rms deviation of fitted atoms = 0.0036- 7.1556 (0.0029) *x* + 6.8670 (0.0050) *y* - 1.5139 (0.0120) *z* = 1.5526 (0.0051)Angle to previous plane (with approximate e.s.d.) = 11.79 (0.10)* -0.0880 (0.0018) N1 * 0.0273 (0.0020) C2 * 0.0689 (0.0021) C3 * 0.0094 (0.0021) C4 * -0.0354 (0.0024) C5 * -0.0377 (0.0021) C6 * -0.0110 (0.0022) C7 * 0.0624 (0.0021) C8 * 0.0474 (0.0020) C9 * -0.0432 (0.0022) C10Rms deviation of fitted atoms = 0.04913.8481 (0.0098) *x* + 11.4658 (0.0058) *y* + 6.3281 (0.0208) *z* = 11.7536 (0.0044)Angle to previous plane (with approximate e.s.d.) = 77.79 (0.06)* -0.0067 (0.0020) C28 * 0.0001 (0.0020) C29 * 0.0072 (0.0023) C30 * -0.0080 (0.0025) C31 * 0.0013 (0.0023) C32 * 0.0060 (0.0021) C27Rms deviation of fitted atoms = 0.00582.0501 (0.0054) *x* + 12.1910 (0.0019) *y* + 5.3371 (0.0154) *z* = 11.4565 (0.0058)Angle to previous plane (with approximate e.s.d.) = 12.28 (0.08)* -0.0704 (0.0021) N17 * 0.0393 (0.0024) C18 * 0.0498 (0.0028) C19 * -0.0036 (0.0029) C20 * -0.0280 (0.0030) C21 * -0.0253 (0.0030) C22 * 0.0012 (0.0029) C23 * 0.0474 (0.0026) C24 * 0.0300 (0.0024) C25 * -0.0404 (0.0026) C26Rms deviation of fitted atoms = 0.03902.0139 (0.0103) *x* + 0.5046 (0.0150) *y* + 17.5147 (0.0030) *z* = 5.5443 (0.0128)Angle to previous plane (with approximate e.s.d.) = 78.01 (0.09)* 0.0047 (0.0018) C38 * 0.0079 (0.0017) N39 * -0.0141 (0.0019) C40 * 0.0073 (0.0022) C41 * 0.0051 (0.0023) C42 * -0.0109 (0.0020) C43Rms deviation of fitted atoms = 0.00902.2942 (0.0112) *x* + 0.5668 (0.0154) *y* + 17.4708 (0.0033) *z* = 5.7456 (0.0097)Angle to previous plane (with approximate e.s.d.) = 1.81 (0.06)* -0.0023 (0.0014) N33 * 0.0007 (0.0014) N34 * 0.0010 (0.0015) C35 * -0.0024 (0.0015) N36 * 0.0029 (0.0015) C37Rms deviation of fitted atoms = 0.00203.5283 (0.0116) *x* - 0.8562 (0.0173) *y* + 16.6404 (0.0084) *z* = 5.4129 (0.0060)Angle to previous plane (with approximate e.s.d.) = 11.00 (0.10)* -0.0031 (0.0021) C44 * 0.0013 (0.0022) C45 * -0.0006 (0.0025) C46 * 0.0019 (0.0027) C47 * -0.0038 (0.0027) C48 * 0.0044 (0.0024) C49Rms deviation of fitted atoms = 0.0029
Refinement. Refinement of *F*^2^ against ALL reflections. The weighted *R*-factor *wR* and goodness of fit *S* are based on *F*^2^, conventional *R*-factors *R* are based on *F*, with *F* set to zero for negative *F*^2^. The threshold expression of *F*^2^ > σ(*F*^2^) is used only for calculating *R*-factors(gt) *etc*. and is not relevant to the choice of reflections for refinement. *R*-factors based on *F*^2^ are statistically about twice as large as those based on *F*, and *R*- factors based on ALL data will be even larger.

### Fractional atomic coordinates and isotropic or equivalent isotropic displacement parameters (Å^2^)

**Table:**

	*x*	*y*	*z*	*U*_iso_*/*U*_eq_	
Ir	0.479659 (11)	0.724128 (9)	0.242431 (7)	0.01435 (3)	
N1	0.5063 (2)	0.76795 (18)	0.12298 (13)	0.0164 (5)	
C2	0.5821 (3)	0.8582 (2)	0.09770 (16)	0.0183 (6)	
C3	0.6254 (3)	0.8918 (2)	0.01826 (17)	0.0241 (7)	
H3	0.6797	0.9550	0.0022	0.029*	
C4	0.5898 (3)	0.8342 (2)	−0.03560 (17)	0.0250 (7)	
H4	0.6227	0.8551	−0.0889	0.030*	
C5	0.5035 (3)	0.7430 (2)	−0.01136 (16)	0.0224 (6)	
C6	0.4578 (3)	0.6834 (3)	−0.06484 (17)	0.0274 (7)	
H6	0.4897	0.7018	−0.1185	0.033*	
C7	0.3680 (3)	0.5992 (3)	−0.03966 (18)	0.0283 (7)	
H7	0.3403	0.5578	−0.0757	0.034*	
C8	0.3166 (3)	0.5737 (2)	0.03924 (17)	0.0253 (7)	
H8	0.2504	0.5173	0.0559	0.030*	
C9	0.3608 (3)	0.6295 (2)	0.09300 (16)	0.0198 (6)	
H9	0.3251	0.6114	0.1463	0.024*	
C10	0.4586 (3)	0.7129 (2)	0.06896 (16)	0.0184 (6)	
C11	0.6096 (3)	0.9190 (2)	0.15802 (16)	0.0189 (6)	
C12	0.5580 (3)	0.8711 (2)	0.23424 (17)	0.0190 (6)	
C13	0.5710 (3)	0.9308 (2)	0.29458 (17)	0.0226 (6)	
H13	0.5371	0.9019	0.3464	0.027*	
C14	0.6325 (3)	1.0308 (2)	0.27935 (19)	0.0284 (7)	
H14	0.6397	1.0695	0.3210	0.034*	
C15	0.6835 (3)	1.0757 (2)	0.20527 (19)	0.0293 (7)	
H15	0.7257	1.1444	0.1961	0.035*	
C16	0.6729 (3)	1.0201 (2)	0.14405 (18)	0.0261 (7)	
H16	0.7085	1.0504	0.0928	0.031*	
N17	0.4354 (3)	0.70179 (19)	0.36314 (13)	0.0239 (6)	
C18	0.2901 (3)	0.7236 (3)	0.38966 (19)	0.0348 (8)	
C19	0.2355 (5)	0.6990 (3)	0.4688 (2)	0.0534 (11)	
H19	0.1330	0.7126	0.4857	0.064*	
C20	0.3248 (5)	0.6572 (3)	0.5199 (2)	0.0562 (11)	
H20	0.2855	0.6390	0.5727	0.067*	
C21	0.4788 (5)	0.6398 (3)	0.49591 (18)	0.0439 (9)	
C22	0.5786 (5)	0.6009 (3)	0.5471 (2)	0.0589 (11)	
H22	0.5423	0.5823	0.6002	0.071*	
C23	0.7259 (5)	0.5885 (3)	0.5236 (2)	0.0557 (11)	
H23	0.7916	0.5607	0.5598	0.067*	
C24	0.7820 (4)	0.6177 (3)	0.4441 (2)	0.0431 (9)	
H24	0.8857	0.6111	0.4275	0.052*	
C25	0.6862 (3)	0.6555 (2)	0.39132 (17)	0.0298 (7)	
H25	0.7237	0.6753	0.3385	0.036*	
C26	0.5336 (4)	0.6647 (2)	0.41575 (17)	0.0290 (7)	
C27	0.1995 (3)	0.7753 (3)	0.3323 (2)	0.0338 (8)	
C28	0.2731 (3)	0.7910 (2)	0.25696 (19)	0.0266 (7)	
C29	0.1941 (3)	0.8497 (2)	0.1998 (2)	0.0354 (8)	
H29	0.2412	0.8625	0.1480	0.043*	
C30	0.0488 (4)	0.8890 (3)	0.2181 (3)	0.0552 (12)	
H30	−0.0021	0.9291	0.1788	0.066*	
C31	−0.0228 (4)	0.8707 (3)	0.2923 (3)	0.0645 (14)	
H31	−0.1232	0.8965	0.3039	0.077*	
C32	0.0508 (4)	0.8153 (3)	0.3494 (3)	0.0541 (11)	
H32	0.0019	0.8036	0.4010	0.065*	
N33	0.4299 (2)	0.55788 (18)	0.25419 (12)	0.0145 (5)	
N34	0.3042 (2)	0.50125 (18)	0.27271 (13)	0.0185 (5)	
C35	0.3547 (3)	0.3969 (2)	0.26948 (14)	0.0169 (6)	
N36	0.5032 (2)	0.38198 (18)	0.25027 (12)	0.0169 (5)	
C37	0.5433 (3)	0.4844 (2)	0.24198 (14)	0.0148 (5)	
C38	0.6914 (3)	0.5248 (2)	0.22220 (15)	0.0167 (6)	
N39	0.6947 (2)	0.63400 (18)	0.21885 (12)	0.0153 (5)	
C40	0.8272 (3)	0.6779 (2)	0.20110 (16)	0.0205 (6)	
H40	0.8300	0.7547	0.1968	0.025*	
C41	0.9589 (3)	0.6165 (3)	0.18894 (19)	0.0317 (8)	
H41	1.0507	0.6500	0.1783	0.038*	
C42	0.9549 (3)	0.5056 (3)	0.19247 (19)	0.0334 (8)	
H42	1.0443	0.4616	0.1840	0.040*	
C43	0.8196 (3)	0.4583 (2)	0.20849 (17)	0.0254 (7)	
H43	0.8147	0.3820	0.2100	0.030*	
C44	0.2545 (3)	0.3068 (2)	0.28691 (16)	0.0215 (6)	
C45	0.3072 (3)	0.2027 (2)	0.27066 (17)	0.0258 (7)	
H45	0.4076	0.1904	0.2490	0.031*	
C46	0.2140 (4)	0.1174 (3)	0.2859 (2)	0.0387 (8)	
H46	0.2504	0.0470	0.2744	0.046*	
C47	0.0680 (4)	0.1350 (3)	0.3179 (2)	0.0477 (10)	
H47	0.0040	0.0767	0.3288	0.057*	
C48	0.0154 (4)	0.2382 (3)	0.3340 (2)	0.0472 (10)	
H48	−0.0852	0.2503	0.3554	0.057*	
C49	0.1075 (3)	0.3236 (3)	0.31940 (18)	0.0330 (8)	
H49	0.0706	0.3936	0.3315	0.040*	
C98	0.9808 (4)	1.2544 (3)	0.0450 (2)	0.0480 (10)	
H98	0.8770	1.2866	0.0552	0.058*	
Cl1	1.03734 (9)	1.19846 (8)	0.13387 (6)	0.0509 (2)	
Cl2	0.98185 (9)	1.15510 (8)	−0.01637 (6)	0.0519 (3)	
Cl3	1.09404 (12)	1.35913 (7)	0.00000 (6)	0.0619 (3)	

### Atomic displacement parameters (Å^2^)

**Table:**

	*U*^11^	*U*^22^	*U*^33^	*U*^12^	*U*^13^	*U*^23^
Ir	0.01027 (5)	0.01557 (6)	0.01753 (5)	−0.00124 (4)	0.00080 (4)	−0.00580 (4)
N1	0.0137 (11)	0.0170 (12)	0.0190 (12)	0.0011 (9)	−0.0057 (9)	−0.0016 (10)
C2	0.0145 (14)	0.0177 (15)	0.0219 (15)	0.0030 (11)	−0.0063 (11)	0.0011 (12)
C3	0.0183 (15)	0.0236 (17)	0.0286 (17)	0.0005 (12)	−0.0045 (12)	0.0029 (13)
C4	0.0206 (15)	0.0322 (18)	0.0177 (15)	0.0092 (13)	−0.0024 (12)	0.0046 (13)
C5	0.0157 (14)	0.0289 (17)	0.0224 (15)	0.0077 (12)	−0.0059 (12)	−0.0047 (13)
C6	0.0237 (16)	0.0372 (19)	0.0218 (16)	0.0120 (14)	−0.0072 (12)	−0.0090 (14)
C7	0.0260 (16)	0.0340 (19)	0.0292 (17)	0.0109 (14)	−0.0132 (13)	−0.0161 (15)
C8	0.0193 (15)	0.0284 (17)	0.0321 (17)	0.0038 (13)	−0.0108 (13)	−0.0130 (14)
C9	0.0155 (14)	0.0219 (16)	0.0228 (15)	0.0031 (11)	−0.0061 (11)	−0.0044 (12)
C10	0.0130 (13)	0.0232 (16)	0.0199 (15)	0.0075 (11)	−0.0074 (11)	−0.0057 (12)
C11	0.0114 (13)	0.0179 (15)	0.0274 (16)	0.0023 (11)	−0.0055 (11)	−0.0018 (12)
C12	0.0089 (13)	0.0183 (15)	0.0309 (17)	0.0003 (11)	−0.0028 (11)	−0.0075 (13)
C13	0.0194 (15)	0.0226 (16)	0.0266 (16)	−0.0023 (12)	−0.0029 (12)	−0.0053 (13)
C14	0.0262 (16)	0.0241 (17)	0.0389 (19)	−0.0049 (13)	−0.0101 (14)	−0.0099 (15)
C15	0.0266 (16)	0.0153 (16)	0.048 (2)	−0.0090 (13)	−0.0103 (15)	−0.0008 (14)
C16	0.0210 (15)	0.0228 (16)	0.0330 (18)	−0.0039 (13)	−0.0059 (13)	0.0048 (13)
N17	0.0315 (14)	0.0215 (14)	0.0196 (13)	−0.0124 (11)	0.0068 (10)	−0.0094 (11)
C18	0.0363 (18)	0.0285 (19)	0.0391 (19)	−0.0156 (14)	0.0231 (15)	−0.0230 (15)
C19	0.065 (3)	0.047 (2)	0.046 (2)	−0.025 (2)	0.0348 (19)	−0.027 (2)
C20	0.090 (3)	0.044 (2)	0.031 (2)	−0.031 (2)	0.036 (2)	−0.0190 (18)
C21	0.090 (3)	0.029 (2)	0.0147 (16)	−0.0232 (19)	0.0005 (17)	−0.0069 (15)
C22	0.118 (3)	0.043 (2)	0.0192 (19)	−0.027 (3)	−0.008 (2)	−0.0038 (17)
C23	0.118 (3)	0.031 (2)	0.029 (2)	−0.013 (2)	−0.045 (2)	0.0019 (17)
C24	0.065 (2)	0.036 (2)	0.036 (2)	−0.0128 (18)	−0.0306 (18)	0.0001 (16)
C25	0.049 (2)	0.0257 (17)	0.0184 (16)	−0.0096 (15)	−0.0127 (14)	−0.0035 (13)
C26	0.051 (2)	0.0201 (17)	0.0175 (15)	−0.0127 (15)	0.0003 (14)	−0.0084 (13)
C27	0.0210 (16)	0.0265 (18)	0.057 (2)	−0.0097 (13)	0.0094 (15)	−0.0267 (16)
C28	0.0155 (14)	0.0172 (16)	0.051 (2)	−0.0057 (12)	0.0017 (13)	−0.0216 (15)
C29	0.0188 (15)	0.0224 (17)	0.073 (3)	0.0013 (13)	−0.0158 (16)	−0.0240 (17)
C30	0.0200 (18)	0.036 (2)	0.124 (4)	0.0090 (16)	−0.026 (2)	−0.047 (2)
C31	0.0127 (18)	0.047 (3)	0.145 (4)	0.0018 (16)	−0.002 (2)	−0.062 (3)
C32	0.0250 (18)	0.042 (2)	0.099 (3)	−0.0172 (16)	0.0256 (19)	−0.050 (2)
N33	0.0144 (11)	0.0160 (12)	0.0132 (12)	−0.0018 (9)	0.0017 (9)	−0.0056 (9)
N34	0.0170 (12)	0.0172 (12)	0.0210 (13)	−0.0051 (10)	0.0037 (10)	−0.0062 (10)
C35	0.0227 (14)	0.0170 (14)	0.0109 (13)	−0.0033 (11)	0.0006 (11)	−0.0035 (11)
N36	0.0207 (12)	0.0162 (12)	0.0146 (12)	−0.0002 (9)	−0.0048 (9)	−0.0029 (10)
C37	0.0161 (13)	0.0180 (14)	0.0109 (13)	0.0006 (11)	−0.0036 (10)	−0.0029 (11)
C38	0.0171 (13)	0.0203 (15)	0.0147 (14)	−0.0013 (11)	−0.0057 (11)	−0.0057 (11)
N39	0.0111 (11)	0.0204 (13)	0.0156 (12)	−0.0015 (9)	−0.0023 (9)	−0.0055 (10)
C40	0.0149 (14)	0.0237 (16)	0.0245 (16)	−0.0026 (12)	−0.0010 (12)	−0.0093 (13)
C41	0.0124 (14)	0.0341 (19)	0.051 (2)	−0.0007 (13)	−0.0025 (14)	−0.0148 (17)
C42	0.0149 (15)	0.035 (2)	0.053 (2)	0.0089 (13)	−0.0069 (14)	−0.0177 (17)
C43	0.0198 (15)	0.0235 (17)	0.0356 (18)	0.0044 (12)	−0.0091 (13)	−0.0111 (14)
C44	0.0268 (15)	0.0195 (15)	0.0182 (15)	−0.0054 (12)	−0.0002 (12)	−0.0035 (12)
C45	0.0299 (16)	0.0197 (16)	0.0274 (17)	−0.0029 (13)	−0.0007 (13)	−0.0049 (13)
C46	0.048 (2)	0.0209 (18)	0.046 (2)	−0.0094 (15)	0.0068 (17)	−0.0090 (16)
C47	0.052 (2)	0.029 (2)	0.060 (3)	−0.0240 (17)	0.0233 (19)	−0.0152 (18)
C48	0.045 (2)	0.037 (2)	0.057 (2)	−0.0215 (17)	0.0272 (18)	−0.0181 (19)
C49	0.0357 (18)	0.0235 (17)	0.038 (2)	−0.0119 (14)	0.0150 (15)	−0.0113 (15)
C98	0.0281 (19)	0.041 (2)	0.079 (3)	0.0085 (16)	−0.0137 (19)	−0.021 (2)
Cl1	0.0316 (5)	0.0592 (6)	0.0578 (6)	−0.0033 (4)	0.0045 (4)	−0.0049 (5)
Cl2	0.0398 (5)	0.0402 (6)	0.0808 (8)	−0.0097 (4)	−0.0109 (5)	−0.0188 (5)
Cl3	0.0956 (8)	0.0249 (5)	0.0750 (8)	−0.0077 (5)	−0.0487 (6)	0.0000 (5)

### Geometric parameters (Å, °)

**Table:**

Ir—C28	1.995 (3)	N36—C37	1.336 (3)
Ir—C12	1.997 (3)	C37—C38	1.460 (3)
Ir—N1	2.084 (2)	C38—N39	1.354 (3)
Ir—N17	2.093 (2)	C38—C43	1.389 (4)
Ir—N33	2.129 (2)	N39—C40	1.346 (3)
Ir—N39	2.196 (2)	C40—C41	1.378 (4)
N1—C2	1.353 (3)	C41—C42	1.375 (4)
N1—C10	1.392 (3)	C42—C43	1.388 (4)
C2—C3	1.412 (4)	C44—C49	1.393 (4)
C2—C11	1.457 (4)	C44—C45	1.398 (4)
C3—C4	1.363 (4)	C45—C46	1.387 (4)
C4—C5	1.414 (4)	C46—C47	1.384 (4)
C5—C6	1.413 (4)	C47—C48	1.387 (4)
C5—C10	1.421 (4)	C48—C49	1.381 (4)
C6—C7	1.367 (4)	C98—Cl1	1.753 (4)
C7—C8	1.403 (4)	C98—Cl3	1.755 (4)
C8—C9	1.380 (4)	C98—Cl2	1.760 (3)
C9—C10	1.403 (4)	C3—H3	0.9500
C11—C16	1.402 (4)	C4—H4	0.9500
C11—C12	1.419 (4)	C6—H6	0.9500
C12—C13	1.411 (4)	C7—H7	0.9500
C13—C14	1.382 (4)	C8—H8	0.9500
C14—C15	1.375 (4)	C9—H9	0.9500
C15—C16	1.387 (4)	C13—H13	0.9500
N17—C18	1.361 (4)	C14—H14	0.9500
N17—C26	1.391 (4)	C15—H15	0.9500
C18—C19	1.411 (5)	C16—H16	0.9500
C18—C27	1.447 (5)	C19—H19	0.9500
C19—C20	1.328 (5)	C20—H20	0.9500
C20—C21	1.417 (5)	C22—H22	0.9500
C21—C22	1.387 (5)	C23—H23	0.9500
C21—C26	1.429 (4)	C24—H24	0.9500
C22—C23	1.352 (5)	C25—H25	0.9500
C23—C24	1.428 (5)	C29—H29	0.9500
C24—C25	1.379 (4)	C30—H30	0.9500
C25—C26	1.398 (4)	C31—H31	0.9500
C27—C28	1.394 (4)	C32—H32	0.9500
C27—C32	1.413 (4)	C40—H40	0.9500
C28—C29	1.408 (4)	C41—H41	0.9500
C29—C30	1.386 (4)	C42—H42	0.9500
C30—C31	1.372 (6)	C43—H43	0.9500
C31—C32	1.365 (6)	C45—H45	0.9500
N33—C37	1.339 (3)	C46—H46	0.9500
N33—N34	1.371 (3)	C47—H47	0.9500
N34—C35	1.347 (3)	C48—H48	0.9500
C35—N36	1.354 (3)	C49—H49	0.9500
C35—C44	1.476 (4)	C98—H98	1.0000
			
C28—Ir—C12	89.81 (10)	N39—C38—C43	122.0 (2)
C28—Ir—N1	93.57 (11)	N39—C38—C37	114.3 (2)
C12—Ir—N1	79.82 (10)	C43—C38—C37	123.7 (3)
C28—Ir—N17	79.82 (12)	C40—N39—C38	118.0 (2)
C12—Ir—N17	95.05 (10)	C40—N39—Ir	125.77 (18)
N1—Ir—N17	171.70 (9)	C38—N39—Ir	116.20 (16)
C28—Ir—N33	98.69 (10)	N39—C40—C41	123.0 (3)
C12—Ir—N33	171.11 (9)	C42—C41—C40	118.7 (3)
N1—Ir—N33	102.08 (8)	C41—C42—C43	119.6 (3)
N17—Ir—N33	83.99 (8)	C42—C43—C38	118.6 (3)
C28—Ir—N39	172.20 (9)	C49—C44—C45	119.2 (3)
C12—Ir—N39	96.86 (9)	C49—C44—C35	121.0 (3)
N1—Ir—N39	83.69 (8)	C45—C44—C35	119.9 (3)
N17—Ir—N39	103.50 (9)	C46—C45—C44	120.6 (3)
N33—Ir—N39	74.85 (8)	C47—C46—C45	119.8 (3)
C2—N1—C10	118.8 (2)	C46—C47—C48	119.7 (3)
C2—N1—Ir	114.28 (18)	C49—C48—C47	120.9 (3)
C10—N1—Ir	126.87 (19)	C48—C49—C44	119.9 (3)
N1—C2—C3	121.6 (3)	Cl1—C98—Cl3	110.20 (18)
N1—C2—C11	115.0 (2)	Cl1—C98—Cl2	111.88 (19)
C3—C2—C11	123.3 (3)	Cl3—C98—Cl2	110.3 (2)
C4—C3—C2	120.4 (3)	C4—C3—H3	119.8
C3—C4—C5	119.4 (3)	C2—C3—H3	119.8
C6—C5—C4	121.7 (3)	C3—C4—H4	120.3
C6—C5—C10	119.5 (3)	C5—C4—H4	120.3
C4—C5—C10	118.8 (3)	C7—C6—H6	119.9
C7—C6—C5	120.2 (3)	C5—C6—H6	119.9
C6—C7—C8	120.2 (3)	C6—C7—H7	119.9
C9—C8—C7	120.9 (3)	C8—C7—H7	119.9
C8—C9—C10	120.0 (3)	C9—C8—H8	119.6
N1—C10—C9	120.3 (2)	C7—C8—H8	119.6
N1—C10—C5	120.6 (3)	C8—C9—H9	120.0
C9—C10—C5	119.0 (3)	C10—C9—H9	120.0
C16—C11—C12	121.1 (3)	C14—C13—H13	119.5
C16—C11—C2	124.3 (3)	C12—C13—H13	119.5
C12—C11—C2	114.5 (2)	C15—C14—H14	119.2
C13—C12—C11	116.9 (3)	C13—C14—H14	119.2
C13—C12—Ir	128.0 (2)	C14—C15—H15	120.2
C11—C12—Ir	115.1 (2)	C16—C15—H15	120.2
C14—C13—C12	120.9 (3)	C15—C16—H16	120.1
C15—C14—C13	121.5 (3)	C11—C16—H16	120.1
C14—C15—C16	119.7 (3)	C20—C19—H19	119.4
C15—C16—C11	119.9 (3)	C18—C19—H19	119.4
C18—N17—C26	119.3 (3)	C19—C20—H20	120.0
C18—N17—Ir	112.8 (2)	C21—C20—H20	120.0
C26—N17—Ir	127.88 (19)	C23—C22—H22	119.0
N17—C18—C19	120.8 (3)	C21—C22—H22	119.0
N17—C18—C27	115.7 (3)	C22—C23—H23	120.2
C19—C18—C27	123.5 (3)	C24—C23—H23	120.2
C20—C19—C18	121.2 (4)	C25—C24—H24	119.9
C19—C20—C21	120.0 (3)	C23—C24—H24	119.9
C22—C21—C20	122.7 (4)	C24—C25—H25	120.1
C22—C21—C26	118.7 (4)	C26—C25—H25	120.1
C20—C21—C26	118.6 (3)	C30—C29—H29	119.5
C23—C22—C21	122.0 (4)	C28—C29—H29	119.5
C22—C23—C24	119.5 (3)	C31—C30—H30	119.6
C25—C24—C23	120.2 (4)	C29—C30—H30	119.6
C24—C25—C26	119.9 (3)	C32—C31—H31	120.1
N17—C26—C25	120.6 (3)	C30—C31—H31	120.1
N17—C26—C21	119.8 (3)	C31—C32—H32	119.9
C25—C26—C21	119.6 (3)	C27—C32—H32	119.9
C28—C27—C32	120.7 (3)	N39—C40—H40	118.5
C28—C27—C18	114.9 (3)	C41—C40—H40	118.5
C32—C27—C18	124.2 (3)	C42—C41—H41	120.7
C27—C28—C29	117.3 (3)	C40—C41—H41	120.7
C27—C28—Ir	115.4 (2)	C41—C42—H42	120.2
C29—C28—Ir	127.3 (2)	C43—C42—H42	120.2
C30—C29—C28	120.9 (4)	C42—C43—H43	120.7
C31—C30—C29	120.9 (4)	C38—C43—H43	120.7
C32—C31—C30	119.8 (3)	C46—C45—H45	119.7
C31—C32—C27	120.3 (4)	C44—C45—H45	119.7
C37—N33—N34	106.6 (2)	C47—C46—H46	120.1
C37—N33—Ir	117.48 (17)	C45—C46—H46	120.1
N34—N33—Ir	135.89 (16)	C46—C47—H47	120.1
C35—N34—N33	103.7 (2)	C48—C47—H47	120.1
N34—C35—N36	114.8 (2)	C49—C48—H48	119.6
N34—C35—C44	121.9 (2)	C47—C48—H48	119.6
N36—C35—C44	123.3 (2)	C48—C49—H49	120.1
C37—N36—C35	100.9 (2)	C44—C49—H49	120.1
N36—C37—N33	113.9 (2)	Cl1—C98—H98	108.1
N36—C37—C38	128.8 (2)	Cl3—C98—H98	108.1
N33—C37—C38	117.2 (2)	Cl2—C98—H98	108.1
